# Indolyl-chalcone derivatives trigger apoptosis in cisplatin-resistant mesothelioma cells through aberrant tubulin polymerization and deregulation of microtubule-associated proteins

**DOI:** 10.3389/fonc.2023.1190988

**Published:** 2023-05-25

**Authors:** Sophia Steinlein, Frank Essmann, Amanda Franceschini Ghilardi, Heike Horn, Julia Schüler, Angelika Hausser, Lijun Sun, German Ott, Claudia Kalla

**Affiliations:** ^1^ Dr. Margarete Fischer-Bosch Institute of Clinical Pharmacology, Stuttgart, Germany; ^2^ Department of Clinical Pathology, Robert-Bosch-Krankenhaus, Stuttgart, Germany; ^3^ Department of Clinical Pharmacology, University Hospital, University of Tuebingen, Tuebingen, Germany; ^4^ Robert Bosch Center for Tumor Diseases, Stuttgart, Germany; ^5^ Harvard Medical School, Center for Drug Discovery and Translational Research, Department of Surgery, Beth Israel Deaconess Medical Center, Boston, MA, United States; ^6^ Charles River Germany GmbH, Freiburg, Germany; ^7^ Institute of Cell Biology and Immunology and Stuttgart Research Center for Systems Biology, University of Stuttgart, Stuttgart, Germany

**Keywords:** indolyl-chalcone, CIT-026, CIT-223, apoptosis, microtubules, stathmin, cisplatin resistance, pleural mesothelioma

## Abstract

**Introduction:**

Malignant pleural mesothelioma (MPM) is a neoplasm with dismal prognosis and notorious resistance to the standard therapeutics cisplatin and pemetrexed. Chalcone derivatives are efficacious anti-cancer agents with minimal toxicity and have, therefore, gained pharmaceutical interest. Here, we investigated the efficacy of CIT-026 and CIT-223, two indolyl-chalcones (CITs), to inhibit growth and viability of MPM cells and defined the mechanism by which the compounds induce cell death.

**Methods:**

The effects of CIT-026 and CIT-223 were analyzed in five MPM cell lines, using viability, immunofluorescence, real-time cell death monitoring, and tubulin polymerization assays, along with siRNA knockdown. Phospho-kinase arrays and immunoblotting were used to identify signaling molecules that contribute to cell death.

**Results:**

CIT-026 and CIT-223 were toxic in all cell lines at sub-micromolar concentrations, in particular in MPM cells resistant to cisplatin and pemetrexed, while normal fibroblasts were only modestly affected. Both CITs targeted tubulin polymerization *via* (1) direct interaction with tubulin and (2) phosphorylation of microtubule regulators STMN1, CRMP2 and WNK1. Formation of aberrant tubulin fibers caused abnormal spindle morphology, mitotic arrest and apoptosis. CIT activity was not reduced in CRMP2-negative and STMN1-silenced MPM cells, indicating that direct tubulin targeting is sufficient for toxic effects of CITs.

**Discussion:**

CIT-026 and CIT-223 are highly effective inducers of tumor cell apoptosis by disrupting microtubule assembly, with only modest effects on non-malignant cells. CITs are potent anti-tumor agents against MPM cells, in particular cells resistant to standard therapeutics, and thus warrant further evaluation as potential small-molecule therapeutics in MPM.

## 1 Introduction

Malignant pleural mesothelioma (MPM) is an aggressive and lethal cancer originating from mesothelial cells in the pleura. Surgery combined with radio-chemotherapy has shown benefit in patients presenting with early-stage disease (1), but most MPM are diagnosed in advanced stage and show multi-focal growth at the time of diagnosis. The current treatment standard for advanced MPM - chemotherapy with cisplatin and pemetrexed or raltitrexed - is effective only in 25-30% of patients (2). Despite its poor efficacy, this therapy regimen continues to be recommended in the recent ERS/ESTS/EACTS/ESTRO guidelines, also due to lack of an approved alternative (3).

Targeting of aberrantly activated molecules by specific inhibitors has shown remarkable clinical response in various cancers and may *per se* be a promising strategy to cure MPM. Inhibition of key molecules in MPM pathogenesis, mTOR (4–6) or kinases such as EGFR, VEGFR, MET (7–9), did suppress MPM cell growth in pre-clinical models but was not effective in clinical trials (10–13). In combination therapy, there is a therapeutic benefit in adding the VEGFR inhibitor bevacizumab to cisplatin/pemetrexed (14). An alternative strategy using immune checkpoint inhibitors (anti-PD-1, anti-PD-L1 antibodies) alone or in combination therapy showed promising therapeutic efficacy, achieving objective responses in 19-29% of patients (15–17). Because of our limited understanding of signaling pathways that contribute to the proliferation of MPM, the development of targeted-based precision therapeutics for patients with MPM remains a significant challenge. Therefore, there is still an unmet need for therapeutic agents that efficiently target cell division and induce cell death in MPM cells.

Chalcone derivatives are novel, highly efficacious anti-cancer agents with minimal toxicity (18). This class of compounds bears a chalcone scaffold – a pharmacophore of known therapeutic potentials, two aromatic rings linked through a ketovinyl chain, which can be reactive to electrophiles. Depending on their substituents, the anti-cancer mechanisms of chalcone derivatives include induction of apoptosis, microtubule binding and cell cycle arrest, or enzyme inhibition (18). The capability of chalcones to act on multiple targets that support tumor growth might represent a unique opportunity for the effective treatment of MPM. Recently, Wegiel and colleagues synthesized a new class of chalcone derivatives of indole-tetralone (CITs) (19). These indolyl-chalcones (CIT-026, CIT-214, CIT-223) target stathmin and induce microtubule destabilization, which results in cell death and deceleration of cell proliferation in prostate and lung cancer cell lines (19).

Stathmin (STMN1) is a microtubule destabilizing protein that plays an important role in cell cycle progression, segregation of chromosomes, cell motility and survival (20). STMN1 acts directly on tubulin heterodimers, preventing the formation of microtubules. Phosphorylation of the amino terminus of STMN1 (Ser16, Ser38) reduces the affinity between STMN1 and tubulin heterodimers, thus enabling tubulin polymerization (21). Microtubules form part of the cytoskeleton and are the major constituents of mitotic spindles. During mitosis, accurate microtubule dynamics is necessary for successful cell cycle progression and tight regulation of STMN1 phosphorylation is crucial for this process. In addition, STMN1 has a “relay” function in signal transduction, integrating multiple signaling pathways: PI3K, MAPK, cAMP, cyclin-dependent kinase (CDK), and Ca^2+/^Calmodulin signaling control the stability and phosphorylation status of STMN1 (20). Up-regulation of STMN1 in cancer cells promotes cell proliferation, migration, metastasis and resistance to chemotherapy. Knockdown of STMN1 significantly reduces tumor growth and metastasis and induces apoptosis in preclinical experiments (22). A first study on STMN1 expression in MPM detected increased protein levels in 7 of 8 MPM tumor samples (23), and knockdown of STMN1 in MPM cells led to inhibition of cell proliferation and motility (24).

Taking into account the chalcones’ capability to act on multiple molecules, we hypothesized that CITs might not only target stathmin but also other microtubule-associated proteins that are deregulated in cancer cells, such as collapsin response mediator protein-2 (CRMP2, also known as DPYSL2). CRMP2 binds to tubulin dimers and promotes microtubule assembly by adding tubulin dimers to the growing microtubule (25). Several signaling pathways, e.g. Sema3A, Rho, PI3K/AKT signaling, regulate CRMP2 phosphorylation and thus its affinity to bind tubulin (26). CRMP2 participates in neuronal growth, vesicle transport, migration and mitosis. Elevated expression of nuclear phosphorylated CRMP2 has been implicated in cancer progression (26, 27). CRMP2 silencing induced tumor cell apoptosis, pointing to CRMP2 as a new potential target for cancer therapy (27).

In this study, we analyzed indolyl-chalcones CIT-026 and CIT-223 for their anti-tumor activity on MPM cells, some of them resistant to standard chemotherapy. We have investigated the drugs’ mechanisms of action to induce mesothelioma cell death, in particular their impact on microtubule dynamics and microtubule regulators STMN1 and CRMP2.

## 2 Materials and methods

### 2.1 Cell lines

MPM cell line NCI-H2052 (sarcomatoid MPM) was purchased from LGC Standards GmbH (Wesel, Germany), MPM cell line MSTO-211H (biphasic MPM) was purchased from DSMZ (Deutsche Sammlung von Mikroorganismen und Zellkulturen, Braunschweig, Germany). MPM patient-derived xenograft (PDX) cell lines PXF698, PXF1118 (both epithelioid MPM) and PXF1752 (sarcomatoid MPM) were obtained from Charles River Germany (Freiburg, Germany). All cell lines were cultured in RPMI medium supplemented with 10% FBS (Sigma-Aldrich, München, Germany). Normal fibroblast cells were gained from reactive lymph node specimens from two individuals as described previously and cultured in RPMI supplemented with 20% FBS (28).

### 2.2 Antibodies and reagents

Antibodies to detect STMN1 (D1Y5A, #13655), phospho-STMN1 (Ser16, #3353), CRMP2 (D8L6V, #35672), phospho-CRMP2 (Thr514, #9397), PARP (46D11, #9532), c-Jun (60A8, #9165), phospho-c-Jun (Ser63, #91952), AKT (C67E7, #4691), phospho-AKT (Ser473, D9E, #4060), ERK1/2 (137F5, #4695), phospho-ERK1/2 (Thr202/Tyr204, #4370), RPS6 (5G10, #2217), phospho-RPS6 (Ser235/236, #4858) were from Cell Signaling (Danvers, MA, USA). Anti-β-Actin (AC-15, A5441) and Anti-α-Tubulin (DM1a, MABT205) were from Sigma-Aldrich.

CIT compounds CIT-026 and CIT-223 were synthesized by the aldol condensation between a tetralone and an indole aldehyde as described previously (19). CITs were dissolved in DMSO at 10 mM and then diluted in culture medium at 0.01-10 µM final concentrations. Paclitaxel was purchased from Selleckchem (Houston, TX, USA).

### 2.3 Immunohistochemistry

IHC was accomplished on sections of FFPE-embedded cell lines using conventional DAB staining on a semi-automated autostainer (LabVision 720, Thermo Fisher Scientific, Waltham, MA, USA) as described earlier (29). After heat-induced epitope retrieval at pH6, anti-Stathmin (1:100) or anti-CRMP2 (1:100) was incubated for 30 minutes. Stathmin and CRMP2 expression were assessed by estimating the intensity of cytoplasmic staining: 0/negative, no staining; 1+, faint; 2+, moderate; 3+, strong staining intensity in at least 10% tumor cells.

### 2.4 Cell viability assay

Cytotoxicity was assessed using a 3-(4,5-dimethylthiazol-2-yl)-2,5-diphenyl tetrazolium bromide (MTT)-based assay (30). Cells were seeded into 96-well plates (4x10^3^ cells/100µl) and incubated with CITs (0.01-3 µM) or DMSO in quintuplicates for 72 h. After addition of 5 mg/ml MTT to each well, the cells were further incubated for 2 h. The produced formazan blue was dissolved in 15% SDS in dimethylformamide-water (1:1), and the absorbance was measured at 550 nm using an EnSpire Multimode Plate Reader 2300 (PerkinElmer). Percent viability was calculated by normalizing absorbance values to those from cells grown in media without drug after background subtraction. IC50 values were calculated by non-linear regression analysis (Prism Software, GraphPad).

### 2.5 Cell death

For real-time investigation of apoptosis and necrosis, cells were seeded at densities of 1x10^4^ cells in 50 µl media into white 96-well plates in triplicates and incubated for 24 hours at 37°C. CITs (1µM), paclitaxel (500 nM) or DMSO (0.01%) were added followed by immediate addition of Real Time-Glo Annexin V Apoptosis and Necrosis reagent (part# JA1011, Promega, Madison, WI, USA) (31). Luminescence and fluorescence signals were monitored overtime up to 56 h using the EnSpire Multimode Plate Reader 2300 (PerkinElmer). Phase-contrast microscopy was used to investigate morphological changes. Cells (5x10^5^ cells/2ml) were seeded into 6-well plates and incubated with CIT-026 (1 µM) or CIT-223 (1 µM) for 24 h. Cells were imaged using a CKX41 inverted microscope and Olympus Cell^F (Olympus Life Science, Tokyo, Japan).

### 2.6 Tubulin polymerization assay

A tubulin polymerization assay kit (part# BK006P, Cytoskeleton, Denver, CO, USA) adapted from the original method of Shelanski et al. and Lee et al. (32, 33) was used to address the direct effects of chemical compounds on tubulin polymerization dynamics. A porcine >99% pure tubulin preparation (3 mg/ml) free of microtubule-associated proteins in 80 mM PIPES pH 6.9, 2 mM MgCl_2_, 0.5 mM EGTA, 1 mM GTP, and 10.2% glycerol was incubated with CIT-026 (100 µM), CIT-223 (100 µM), paclitaxel (10 µM), or buffer in duplicate samples. Microtubule polymerization was initiated by tubulin addition; changes in microtubule turbidity were measured kinetically at 340 nm at 35°C (EnSpire Multimode Plate Reader 2300, PerkinElmer).

### 2.7 Immunofluorescence

Analysis by immunofluorescence microscopy was performed as described earlier (34). Briefly, 1x10^5^ cells/cm^2^ cells were seeded on #1 coverslips and incubated with CITs (100 nM), paclitaxel (100 nM) or DMSO (0.001%) for 24 h. Then, cells were fixed with ice-cold methanol/acetone (1:1) for 20 min and subsequently incubated in IF buffer (4% bovine serum albumin, 0.05% saponin in PBS) for 1 h. Coverslips were incubated in IF buffer containing α-Tubulin antibody (1:500) overnight at 4°C. Thereafter, cells were washed twice in IF buffer and incubated with Alexa594-coupled chicken anti-mouse antibody (Fisher Scientific, Schwerte, Germany, # A-21201; 1:400). DNA was detected by incubation in a 4,6-diamidino-2-phenylindole solution (DAPI, 1 µg/ml; Sigma). Fluorescence images were captured using a Leica TCS SP8 confocal laser scanning microscope equipped with an HCX PL APO x 63 immersion objective (Leica, Wetzlar, Germany) at wavelengths ex(405 nm)/em(408-475 nm) and ex(552 nm)/em(560-637 nm). Images are projections of recorded z-stacks of various dimensions. The mitotic index was estimated using an epifluorescence microscope (Leica DMRA), x 40 objective. The mitotic index corresponded to the relative number of mitotic nuclei among at least 500 nuclei per sample (visualized by DAPI staining) and is expressed as percentage.

### 2.8 Immunoblotting

Snap-frozen cells were homogenized in lysis buffer (50mM TRIS-HCl pH 7.6, 250mM NaCl, 5mM EDTA, 0,1% Triton X-100) containing cOmplete protease inhibitor cocktail and PhosSTOP phosphatase inhibitor (Roche, Mannheim, Germany) by sonication. Immunoblotting was performed as previously described (35). Antibodies were used at 1:1.000 dilution, except antibodies against CRMP2, phospho-Stathmin (1:500); phospho-AKT, phospho-ERK1/2, phospho-RPS6 (1:2.000); β-Actin (1:10.000). Luminescent signals were detected with the digital gel documentation system Stella3200 (Raytest, Straubenhardt, Germany) and quantified using ImageJ 152-win-java8 software (National Institutes of Health, USA).

### 2.9 Human phospho-kinase array

A human phospho-kinase antibody array (part# ARY003B, R&D Systems) was used for profiling the relative site-specific phosphorylation in several kinases and kinase substrates, according to the manufacturer’s instructions. Cells were seeded in T25 flasks (4x10^5^ cells/ml) and treated with 1 µM CIT-026 or 0.01% DMSO for 1 h, 4 h and 24 h. Cell lysates were prepared with lysis buffer provided with the kit. Phosphokinase array membranes were incubated with 200 µg of protein extract in each condition overnight at 4°C. Following a washing step, membranes were incubated with provided secondary antibodies at room temperature for 2 h and then exposed to streptavidin coupled to horseradish peroxidase at room temperature for 20 min. Luminescent signals were detected with a CCD Camera Stella3200 (Raytest, Straubenhardt, Germany). Spots were quantified with the ImageJ software.

### 2.10 STMN1 knockdown

Cells were transfected with either 30 nM non-targeting control siRNA (ON-TARGETplus Non-targeting pool, Horizon Discovery, Cambridge, United Kingdom) or 30 nM human pooled STMN1 siRNAs (ON-TARGETplus Human STMN1 siRNA, Horizon Discovery) using Lipofectamine 3000 (Thermo Fisher Scientific) according to the manufacturer’s instructions (36). Twenty-four hours after transfection, cells were treated with CIT-026 (0.3-3µM) in 96-well plates for 72 h or 96 h and subsequently subjected to MTT assay. To confirm successful knockdown, STMN1 and phospho-STMN1 expression was analyzed by immunoblotting.

### 2.11 Molecular modeling

The crystal structure of tubulins bound to plinabulin was retrieved from the Protein Data Bank (PDB ID: 5C8Y) (37). The proteins were separated from the ligand and submitted to the Protein Preparation Workflow^2^ (Schrödinger Suite Release 2022-3) to add missing side chains, optimize hydrogen atoms within the OPLS4 force field (38), assign residues protonation at pH 7.4 using PROPKA, and remove water molecules. The chemical structures of CIT-223 and CIT-026 were drawn in the 2D Sketcher in Maestro 13.3.121 (Schrödinger Release 2022-3). Further refinement of ligand structure was conducted with LigPrep (Schrödinger Release 2022-3), including an energy minimization with the OPLS4 force field. The co-crystallized ligand plinabulin was used to center the grid box for docking of CIT-026 and CIT-223, generated by the Receptor Grid Generation (Schrödinger Release 2022-3). Ligand docking was conducted using Glide Standard Precision (SP) (Schrödinger Release 2022-3). Visualization of protein-ligand interactions and the generation of corresponding figures were performed with ChimeraX (UCSF version 1.3) (39).

### 2.12 Statistical analysis

The statistical tests were done using GraphPad Prism 5.00. The test used, number of samples and independent experiments, and significance are indicated in the Methods and respective figure legends.

## 3 Results

### 3.1 CIT-026 and CIT-223 decrease MPM cell viability and proliferation

**Figure 1:**
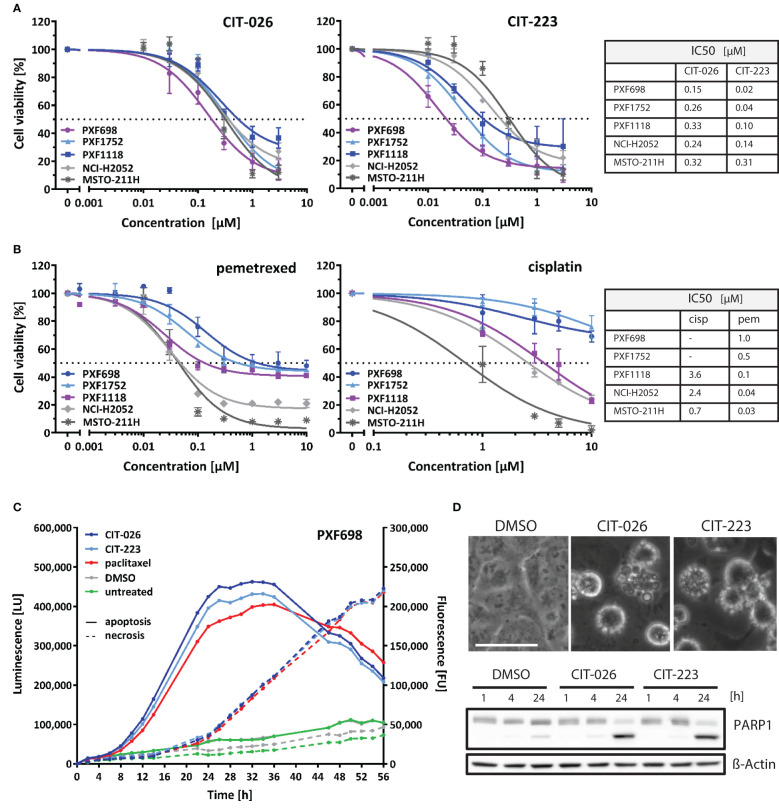
CITs induce concentration-dependent decrease of mesothelioma cell viability. **(A, B)** MPM cell lines were exposed to the indicated concentrations of CIT-026, CIT-223, pemetrexed or cisplatin. The viability of the cells was measured after 72 h of treatment by the MTT assay. Non-linear regression analysis of log(inhibitor) versus response was used for IC50 calculation. Lines, mean ± SD of independent triplicate experiments. **(C)** PXF698 cells were exposed to CIT-026 (1 µM), CIT-223 (1 µM), paclitaxel (500 nM) or DMSO in the presence of the RealTime-Glo Annexin V Apoptosis and Necrosis Assay. Luminescence (phosphatidyl:annexin V binding – apoptosis; solid line) and fluorescence (loss of membrane integrity – necrosis; dashed line) were recorded for 56 h at the indicated time points. A clear temporal lag between phosphatidylserine exposure and lack of membrane integrity is indicative of apoptotic cell death leading to secondary necrosis. Data shown as mean of three replicate samples for each treatment, representative of two independent experiments. **(D)** Apoptotic membrane blebbing and PARP1 cleavage at 24 h treatment of PXF698 cells with 1 µM CIT-026 or 1 µM CIT-223, detected by phase-contrast microscopy and immunoblotting. Scale, 50 µm.

We investigated the cytotoxic efficacy of indolyl-chalcones CIT-026 and CIT-223 on two conventional MPM cell lines (NCI-H2052, MSTO-211H) and three MPM patient-derived xenograft (PDX) cell lines (PXF698, PXF1118, PXF1752). MPM cells were exposed to increasing concentrations (0.01-3.0 µM) of CIT-026 and CIT-223 for 72 hours, after which cell viability was assessed by an MTT assay. Both CITs strongly impaired cell viability and proliferation in all five cell lines at sub-micromolar concentrations (IC50: 0.15-0.33 µM CIT-026, 0.02-0.31 µM CIT-223) (**Figure 1A**).

Notably, the CITs were highly effective in all three MPM cell lines with poor chemotherapy response: PXF698 and PXF1752 cells were resistant to cisplatin and partly resistant to pemetrexed (**Figure 1B**), but sensitive to CIT-026 and the best responders to CIT-223 (IC50 0.02-0.04 µM); PXF1118 cells were sensitive to high concentrations of cisplatin only and partly resistant to pemetrexed (**Figure 1B**), but responded well to CIT-026 (IC50 0.33 µM) and CIT-223 (IC50 0.10 µM).

In addition, we analyzed the impact of CIT-026 and CIT-223 on the viability and proliferation of non-tumor cells, i.e. fibroblasts from two individuals. MTT assay did not indicate reduced viability of fibroblasts after 72 h incubation with 0.3 µM CIT-026 or CIT-223, a concentration that reduced viability of all five MPM cell lines (**Supplementary Figure 1**). At 3 µM, the effects of both CITs on fibroblasts were comparable to the responses obtained with 0.03-0.3 µM CIT-223 or 0.1-0.3 µM CIT-026 in MPM cells indicating that the impact of the CITs on cell viability was at least ten times stronger on tumor cells than on non-tumor cells (**Figure 1A**; **Supplementary Figure 1**).

### 3.2 CIT-026 and CIT-223 induce apoptotic cell death in MPM cells

To identify the mechanism of cell death induced by CIT compounds, we performed a real-time assay that measured the exposure of phosphatidylserine on the outer leaflet of the plasma membrane by annexin V binding as well as the loss of membrane integrity by cellular uptake of a fluorescent membrane-impermeable DNA stain. Apoptotic cell death is characterized by a delay between phosphatidylserine translocation and the loss of membrane integrity, whereas necrotic cell death is defined by concomitant annexin V binding and loss of membrane integrity. We found that CIT-026 (1 µM) and CIT-223 (1 µM) induced an apoptotic signature in PXF698 and PXF1752 cells with a clear temporal lag between phosphatidylserine exposure and loss of membrane integrity of 8-12 hours indicating apoptotic cell death (**Figure 1C**). As a control, we used paclitaxel to induce canonical apoptotic cell death. Induction of apoptosis was confirmed by apoptotic membrane blebbing and cleavage of the caspase-substrate PARP1 (poly (ADP-ribose) polymerase 1) detected in PXF698 and PXF1752 cells following exposure to CIT-026 (1 µM) or CIT-223 (1 µM) for 24 hours (**Figure 1D**).

### 3.3 CIT compounds disturb tubulin polymerization

**Figure 2:**
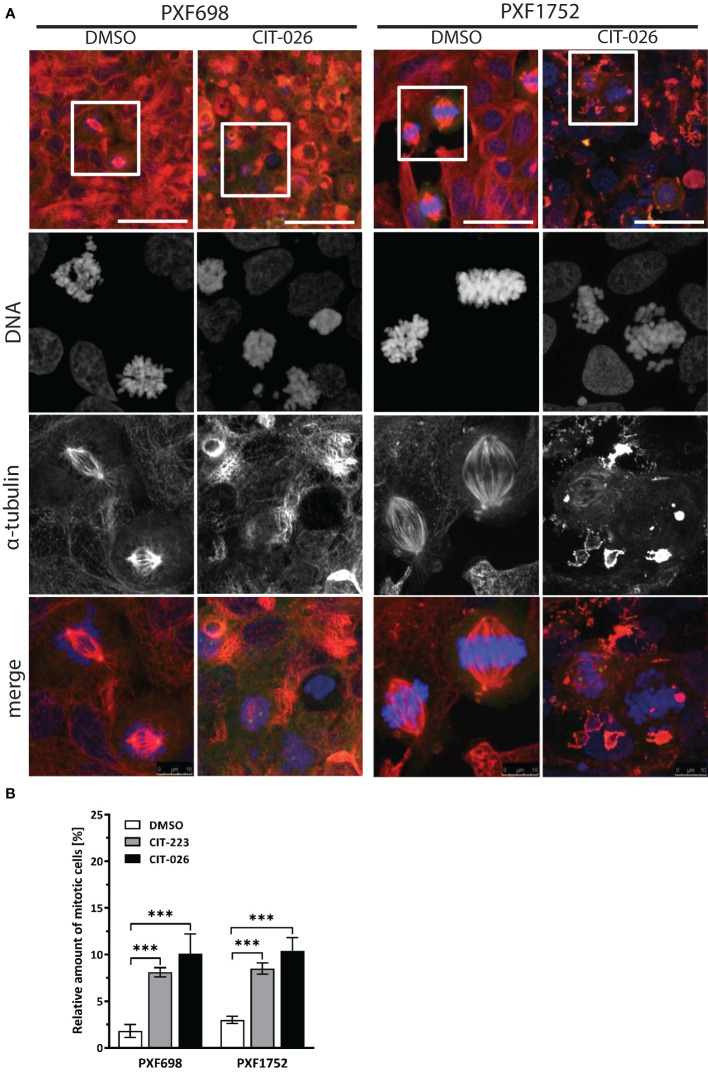
CIT compounds induce abnormal formation of tubulin fibers in mesothelioma cells. **(A)** PXF698 and PXF1752 cells were stained using α-tubulin (red) and DAPI (blue) and analyzed by confocal laser scanning microscopy after treatment with 100 nM CIT-026 or DMSO for 24 h. Scale bar, 50 μm. Representative images of two independent experiments are shown. **(B)** Quantification of the percentage of mitotic nuclei after treatment of PXF698 and PXF1752 cells with CIT-026, CIT-223 or DMSO for 24 h. DNA was stained using DAPI to identify interphase nuclei and assemblies of condensed chromosomes (mitotic nuclei). At least 500 nuclei per sample were scored. Data shown as mean ± SD of three independent areas. Statistical significance was determined by One-Way ANOVA and Tukey’s *post hoc* test, ***P<0.001.

To clarify the effects of CITs on the cellular microtubule network and mitotic spindles in MPM cells, we visualized microtubules after 24 hours of CIT-026 and CIT-223 treatment using an α-tubulin specific antibody. Control DMSO-treated cells had a normal fine-structured filamentous tubulin network and regular mitotic spindles. In contrast, PXF698 cells exposed to CIT-026 or CIT-223 displayed an aberrant microtubule network featuring shortened, branched tubulin fibers that accumulated in clusters (**Figure 2A**). In PXF1752 cells, a complete lack of long fibers was observed upon CIT treatment (**Figure 2A**). In both cell lines, abnormal spindle morphology was accompanied by perturbed chromosomal arrangement in (pro)metaphase and an accumulation of mitotic nuclei, i.e. an 2.8-fold to 5.5-fold increase in the number of mitotic nuclei compared with control cells (**Figure 2B**).

**Figure 3:**
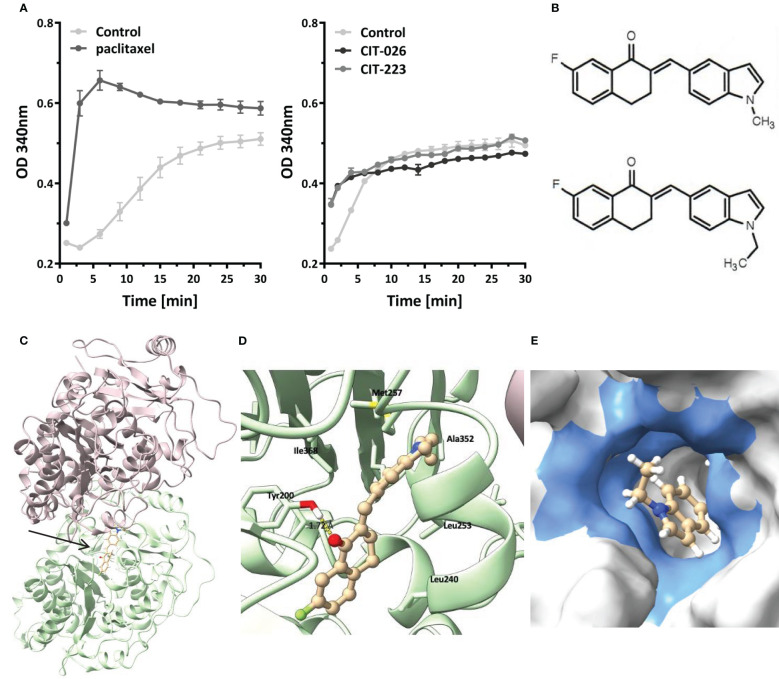
CIT compounds promote *in situ* tubulin polymerization. **(A)** Purified tubulin was incubated with paclitaxel (10 µM), CIT-026 (100 µM), CIT-223 (100 µM) or buffer alone. Microtubule polymerization was measured kinetically at 340 nm at 35°C. Data shown as mean ± SD of two replicate samples for each treatment, representative of two independent experiments. **(B)** The chemical structures of CIT-026 (top) and CIT-223. **(C)** Top docking pose (Glide Gscore: -9.89 kcal/mol) of CIT-223 in the colchicine binding site of β-tubulin. **(D)** Detailed ligand-receptor binding interactions. **(E)** Hydrophobic pocket (colored in blue) surrounding the ethyl-indole moiety. CIT-223 shown as ball and stick in beige, α-tubulin in magenta, β-tubulin in green, oxygen atom in red, nitrogen atom in blue, sulfur atom in yellow, and fluorine atom in bright green, H-bond indicated by yellow dash line.

To determine if the influence of CITs on microtubule formation was due to on-target activity, we examined the direct effect of CIT compounds on tubulin polymerization rates by incubation of purified porcine tubulin with CIT-026 and CIT-223 and compared the results with those of paclitaxel, a well-established microtubule-stabilizing agent. Microtubule formation was measured kinetically at an absorbance of 340 nm. In the presence of paclitaxel, the kinetics of tubulin polymerization was enhanced and the polymerization equilibrium shifted towards polymerized tubulin due to microtubule stabilization (**Figure 3A**). CIT-026 and CIT-223 had a similar effect on the kinetics, but not on the equilibrium of tubulin polymerization seen after 10 min indicating that CITs promote tubulin polymerization, but not microtubule stabilization (**Figure 3A**). These data suggest that direct interaction with tubulin contributes to microtubule disturbance inferred by CIT which ultimately leads to aberrant spindle assembly and mitotic arrest.

We performed molecular modeling studies to support the experimental findings that the CITs directly act on tubulin to elicit apoptosis of MPM cells. The Schrödinger modeling software package was used to simulate the binding interactions between the CITs and tubulins. Docking of the CITs to the colchicine binding site of tubulins (PDB ID: 5C8Y) generated top scoring binding poses showing essentially the same protein-ligand interactions for CIT-223 and CIT-026 (**Figure 3C**; **Supplementary Figure 2A**). The ligands lodged deeply into the colchicine binding site of the β-tubulin subunit, and did not form contact with the α-tubulin subunit. A hydrogen bond (H-bond) interaction was observed for both CIT-026 (bond length: 2.18 Å) and CIT-223 (bond length: 1.72 Å) between the carbonyl oxygen of the tetralone moiety and the side chain phenolic OH of Tyr^200^ (**Figure 3D**; **Supplementary Figure 2B**). The indole moiety was surrounded by hydrophobic residues Ala^314^, Ala^315^, Ile^316^, Leu^246^, Met^257^, and Ala^352^. The bulkier ethyl group in CIT-223 (**Figure 3B**) occupied this hydrophobic cavity more efficiently than the methyl substituent in CIT-026, which resulted in a closer proximity to the side chains of the residues Ile^316^, Met^257^, and Ala^352^ (**Figure 3E**). These enhanced hydrophobic interactions imposed by the ethyl group in CIT-223 might, at least in part, explain its stronger anti-tumor activity than CIT-026.

### 3.4 STMN1 and CRMP2 are targets of CIT compounds

**Figure 4:**
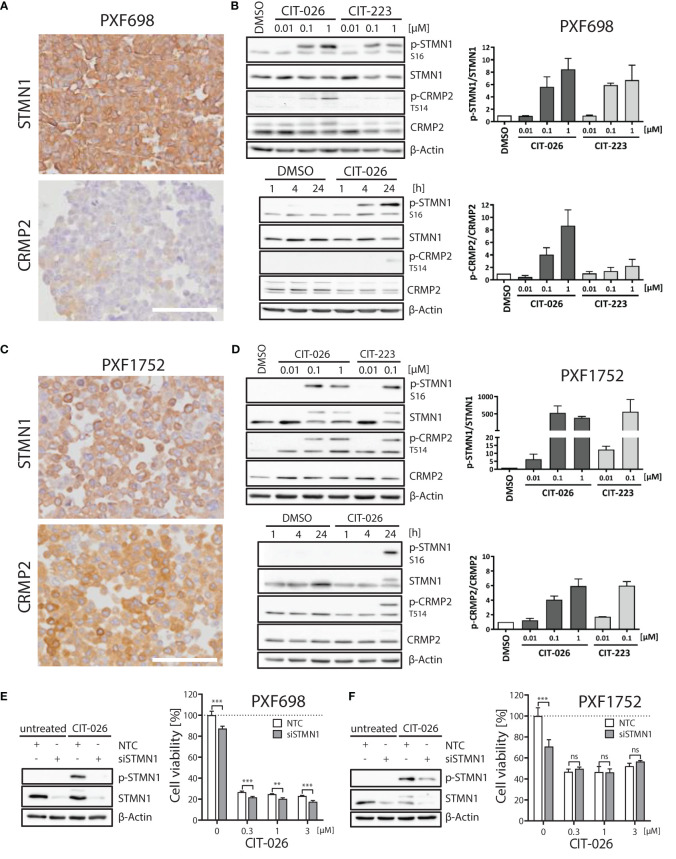
Role of STMN1 and CRMP2 in mediating the anti-tumor effects of CIT-026 and CIT-223. **(A, C)** Both MPM cell lines PXF698 **(A)** and PXF1752 **(C)** expressed high levels of STMN1 whereas significant amounts of CRMP2 were only expressed in PXF1752, determined by immunohistochemistry. Scale bar, 100 µm. **(B, D)** Immunoblotting with STMN1 and CRMP2 specific antibodies of PXF698 **(B)** and PXF1752 cells **(D)** treated with increasing concentrations of CIT-026 or CIT-223 for 24 h (top) or 1 µM CIT-026 or DMSO for 1 h, 4 h and 24 h (bottom). Bar graphs represent semi-quantitative analysis of phospho-protein expression relative to total protein, normalized to protein levels in DMSO treated cells. Mean ± SD of two independent experiments. **(E, F)** PXF698 **(E)** and PXF1752 cells **(F)** were transfected with STMN1-siRNA or non-target control (NTC)-siRNA. Knockdown of STMN1 and suppression of phospho-STMN1 (S16) induction after exposure to CIT-026 (1 µM, 24h) were confirmed by immunoblotting. Cell viability of transfected PXF698 **(E)** and PXF1752 cells **(F)** exposed to the indicated concentrations of CIT-026 for 96h and 72h, respectively, was measured by MTT assay. Mean ± SD of five replicate samples, representative of two independent experiments. Statistical significance was determined by Two-Way ANOVA and Bonferroni posttests, **P<0.01; ***P<0.001¸ ns, P>0.05.

Next, we evaluated the role of STMN1 and CRMP2, two major tubulin and mitosis regulators, in mediating the anti-tumor effects of CITs in MPM cells. All five MPM cell lines expressed STMN1 protein (**Figure 4**; **Supplementary Figure 3**). Significant amounts of CRMP2 were only expressed in PXF1752 and MSTO-211H whereas PXF698 and NCI-H2052 had low-level CRMP2 expression and PXF1118 was negative (**Figure 4**; **Supplementary Figure 3**).

When PXF698 and PXF1752 cells were treated with increasing concentrations of CIT-026 or CIT-223 for 1 to 24 hours, phosphorylation of STMN1 (Ser16) and CRMP2 (Thr514) was effectively enhanced in a dose- and time-dependent manner and reached hyper-phosphorylation levels at 24 hours (**Figures 4B, D**). STMN1 and CRMP2 protein expression remained unaltered during CIT therapy.

To determine whether the activity of CITs was dependent upon STMN1, we examined the consequences of reduced STMN1 protein expression on the efficacy of CIT therapy by knocking down STMN1 in cell lines PXF698 and PXF1752. Knockdown of STMN1 by siRNA was confirmed by immunoblotting (**Figures 4E, F**) and significantly reduced the viability of both MPM cell lines. In PXF698 cells, cell death in response to CIT-026 was accelerated in the absence of STMN1 (**Figure 4E**). In PXF1752 cells, partial STMN1 knockdown did not have a significant impact on the therapeutic effect of CIT-026 (**Figure 4F**). Taken together, these findings suggest that the activity of CITs is associated with STMN1 and CRMP2 hyper-phosphorylation, but is not dependent upon STMN1 activity.

### 3.5 CIT compounds affect intracellular signaling in mesothelioma cells

**Figure 5:**
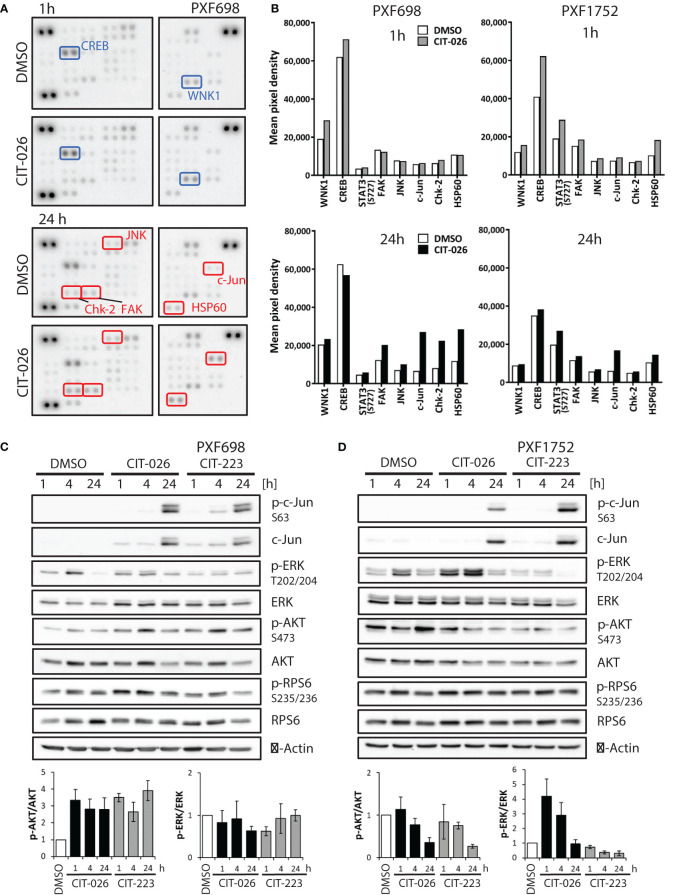
CIT compounds affect intracellular signaling in mesothelioma cells. **(A)** Phospho-kinase array membranes were incubated with protein extracts from PXF698 treated with 1 µM CIT-026 or DMSO alone for 1h and 24 h. The phosphorylation of WNK1 and CREB was increased by CIT-026 after 1 h (blue rectangles); Chk2, c-Jun, FAK, JNK and HSP60 phosphorylation was increased after 24 h CIT-026 treatment (red rectangles). **(B)** Comparison of phospho-kinase array data of PXF698 and PXF1752 treated with 1 µM CIT-026 (1 h – grey bars, 24 h – black bars) or DMSO (white bars). Mean pixel density of duplicate spots. **(C, D)** Immunoblot analysis of signaling molecules in PXF698 **(C)** and PXF1752 cells **(D)** treated with 1 µM CIT-026, 1 µM CIT-223 or DMSO for 1 h, 4 h and 24 h. Bar graphs represent relative p-AKT (S473)/total AKT and p-ERK (T202/204)/total ERK protein levels, normalized to protein levels in DMSO treated cells. Mean ± SD of three independent experiments.

To gain a broader insight into cellular pathways affected by CIT compounds, we conducted an unbiased phospho-kinase array analysis, encompassing 43 distinct kinase phosphorylation sites and two related proteins. A 1-hour CIT-026 treatment of PXF698 and PXF1752 cells enhanced phosphorylation of the microtubule-associated kinase WNK1 and the transcription factor CREB in both cell lines compared to DMSO-treated cells (**Figures 5A, B**; **Supplementary Tables 1, 2**). In more detail, we observed a 50% and 30% increase in phospho-WNK1 in PXF698 and PXF1752, respectively. A 50-60% increase in CREB phosphorylation was detected in PXF1752 after 1 hour and in PXF698 cells after 4 hours therapy.

Upon 24-hour CIT-026 exposure, we observed an activation of proteins involved in mitotic arrest, stress response and apoptosis induction: phospho-Chk2 (Thr68) was enhanced in PXF698 cells, phospho-c-Jun and HSP60 in both cell lines, (**Figures 5A, B**; **Supplementary Tables 1, 2**). Immunoblot analysis showed that c-Jun phosphorylation in response to CIT-026 and CIT-223 was associated with induction of c-Jun expression (**Figures 5C, D**). Corresponding to this finding, phosphorylation of Jun-regulating kinases JNK and FAK was (slightly) enhanced (by 40-60% in PXF698, 20% in PXF1752). CIT compounds did not affect mTOR signaling, indicated by unaltered mTOR and RPS6 phosphorylation levels (**Figures 5C, D**; **Supplementary Tables 1, 2**).

Finally, we examined the effects of CITs on two main effectors of survival and proliferation, AKT and ERK1/2, by immunoblot assays. Interestingly, we observed cell-line dependent differences. In PXF698 cells, CIT treatment increased phospho-AKT (Ser473), but did not affect ERK (Thr202/204) phosphorylation (**Figure 5C**). In PXF1752 cells, both CITs induced time-dependent decrease in phospho-AKT levels. Short-time exposure (1-4 hours) of CIT-026 induced an increase in ERK phosphorylation, which disappeared after 24 hours treatment; CIT-223 reduced phospho-ERK without early phosphorylation induction (**Figure 5D**).

Taken together, our data suggest that CIT compounds act *via* deregulation of tubulin-associated phospho-proteins and kinases (STMN1, CRMP2, WNK1) and affect key regulators of mitotic arrest (Chk2), stress response (JNK/c-Jun, HSP60, FAK), cell survival and apoptosis induction (AKT, ERK1/2, JNK/c-Jun).

## 4 Discussion

The present study shows that the novel indolyl-chalcones CIT-026 and CIT-223 act as microtubule-targeting agents to induce mitotic arrest and apoptosis in mesothelioma cell lines *in vitro.* Both CITs were toxic to five mesothelioma cell lines at a sub-micromolar concentration, comparable to their efficacy in prostate and lung cancer cells observed by Wegiel et al. in their first *in vitro* study of CIT compounds (19).

**Figure 6:**
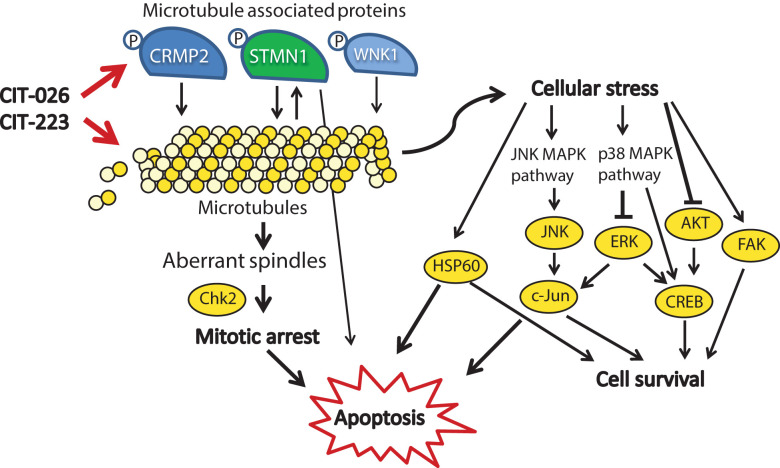
Mechanistic scenario of the molecular action of CIT-026 and CIT-223. CIT compounds promote the assembly of aberrant microtubule fibers *via* direct interaction with tubulin and phosphorylation of microtubule regulatory proteins STMN1, CRMP2 and WNK1. Formation of aberrant tubulin fibers induces abnormal spindle morphology, mitotic arrest and apoptosis. Apoptosis is mediated by activation of pro-apoptotic signaling (Chk2, STMN1, JNK/c-Jun, HSP60) and inhibition of AKT/ERK, thus counteracting stress-induced pro-survival signaling (HSP60, c-Jun, CREB).

In addition to the demonstration of anti-tumor efficacy, our results provide new insights into the mechanism of action of CIT compounds. Using a tubulin polymerization assay, we found that CIT-026 and CIT-223 directly interact with tubulin molecules and accelerate tubulin polymerization. Like other indolyl-chalcones that interact with tubulin (40, 41), CITs cause apoptosis as a result of disruption of mitotic progression due to abnormal spindle morphology (**Figure 6**). Consistently, we observed an accumulation of (pro)metaphase nuclei and activation of Chk2, which promotes cell cycle arrest and apoptosis (42). Moreover, computational studies indicate that the CITs form high affinity complex with β-tubulin by occupying its colchicine binding site, and both H-bonding and hydrophobic interactions contribute to ligand-protein stabilization.

Treatment with CITs also resulted in activation of proteins involved in stress response and apoptosis, including transcription factors (CREB, c-Jun), kinases (JNK, FAK) and protein chaperonin HSP60. Induction of c-Jun, CREB and FAK is known to promote survival of cells upon cellular stress and pro-apoptotic stimuli (43–45). HSP60 shows both pro-survival and pro-death functions, depending on differential interactions with caspase-3 (46). Similar to our observations, treatment with the microtubule-stabilizing agent paclitaxel resulted in activation of JNK and c-Jun, which forced apoptotic cell death (47). In addition, several reports show that apoptosis induction in response to microtubule-targeting agents, such as paclitaxel, natural and synthetic chalcones, is executed *via* inhibition of AKT/ERK signaling (48–50). Consistently, apoptosis induction upon CIT treatment was associated with AKT inhibition and ERK1/2 inhibition in PXF1752 cells: phospho-AKT was reduced by both CITs, phospho-ERK by CIT-223. In contrast, CIT-026 induced a transient activation of ERK1/2 in PXF1752 cells, and phospho-AKT was enhanced upon CIT treatment in PXF698 cells, which however did not prevent apoptosis induction. We conclude that apoptosis of mesothelioma cells in response to CIT treatment is mediated by activation of pro-apoptotic signaling (JNK, HSP60) and – depending on the cellular background – inhibition of AKT/ERK, thus overriding pro-survival signaling by c-Jun, CREB and FAK (**Figure 6**).

Unlike conventional microtubule-targeting agents, small-molecule chalcones are not only able to interact with tubulin, but can also bind to various enzymes including oxidoreductases, transcription factor NF-κB (51), and pro-apoptotic proteins (e.g. caspases-3 and -8) (41), depending on their substituents (18). Chalcone interaction with tubulin and enzymes is mediated by the formation of hydrogen bonds and hydrophobic interactions between the chalcone-substituents and tubulin (34, 46) or amino acids present in the enzyme active sites (41). Here, we observed hyper-phosphorylation of the microtubule regulators STMN1 and CRMP2 in response to CIT treatment. Mechanistically, STMN1 and CRMP2 hyper-phosphorylation might be due to direct interaction of the indolyl-chalcones with phosphorylation sites of both phosphoproteins – in conjunction with enhanced phosphorylation by the kinase JNK. JNK is one of the kinases that regulate STMN1 activity by phosphorylation (22, 47, 52), and JNK was activated itself by CIT treatment. Alternatively, hyper-phosphorylation of STMN1 might be an effect of forced tubulin polymerization as detected in Xenopus eggs (53).

In any case, it is conceivable that STMN1 and CRMP2 hyper-phosphorylation adds to the direct effect of CITs on microtubule polymerization (1): CRMP2 stabilizes polymerized tubulin at the plus end of microtubules and phosphorylated CRMP2 loses its affinity for microtubule heterodimers (25). Hence, CRMP2 hyper-phosphorylation is thought to destabilize microtubules (2). STMN1 binds tubulin heterodimers, thus preventing the formation of microtubules. Phosphorylation of STMN1 reduces the affinity of STMN1 and tubulin heterodimers (21). Hence, STMN1 hyper-phosphorylation promotes tubulin polymerization. Simultaneously reduced tubulin stability (pCRMP2) and augmented polymerization (pSTMN1) is expected to interfere with the accurate formation of microtubule structures that are necessary for successful cell cycle progression (**Figure 6**). Accordingly, MPM cells displayed an aberrant microtubule network consisting of shortened, branched tubulin fibers upon CIT treatment.

The activity of tubulin-associated proteins is not necessarily restricted to microtubule polymerization. Instead, STMN1 and the microtubule-associated kinase WNK1 are also involved in cell cycle control, cell survival and apoptosis, as components of diverse cancer signaling networks including PI3K/AKT, TNF and NF-κB signaling (20, 22, 54). Accordingly, CIT-induced phosphorylation of STMN1 and WNK1 may affect intracellular signaling and contribute to mitotic arrest and apoptosis. In support of this hypothesis, phosphorylated WNK1 was shown to block insulin-stimulated mitosis (55), and GDP366-induced phosphorylation of STMN1 promoted cell cycle arrest and apoptosis in leukemia cells (56).

Although hyper-phosphorylation of STMN1 and CRMP2 was a major effect of CIT treatment, STMN1 and CRMP2 activity was not essential for anti-cancerous effects of CITs. Knockdown of STMN1 in MPM cells did not block the anti-tumor activity of CIT-026, and STMN1 knockdown in prostate cancer cells did only partially block CIT-induced cell death (19). Furthermore, CITs had a similar efficacy in CRMP2-positive (PXF1752, MSTO-211H) and CRMP2-negative MPM cells (PXF1118, NCI-H2052). Together, our findings suggest that CITs exert a dual mode of action in STMN1/CRMP2-positive cells: direct interaction with tubulin in concert with STMN1/CRMP2 inhibition. In STMN1/CRMP2-negative cells, where STMN1/CRMP2 are *per se* inactive, CITs act efficiently *via* direct interaction with tubulin only. Thus, CITs may be efficient therapeutic agents suitable not only for STMN1/CRMP2-positive tumors.

While both CITs were toxic to all five MPM cell lines at a concentration of 0.3 µM, the viability of normal fibroblasts was not significantly affected at this concentration. At a higher concentration (3 µM), fibroblasts were modestly affected, consistent with the fact that they do proliferate at sub-confluent densities. This finding suggests that a therapeutic window exists wherein lower concentrations of CIT-026 and CIT-223 may decrease the viability of cancer cells, with modest effects on normal cells. Thus, CITs may have advantages over the currently available microtubule-targeting agents, which often induce immunosuppression and neurotoxicity (57).

The majority (70-75%) of MPM tumors do not respond to the current standard and recommended therapeutics cisplatin and pemetrexed (2). In this context, it is important to note that both CIT compounds were highly effective against all MPM cells investigated here, in particular MPM cells that were partially or fully resistant to cisplatin and/or pemetrexed. Other agents that target tubulin, including the marketed drug paclitaxel, have not shown significant therapeutic effect in MPM. While the CIT compounds bind to tubulin, they seem to elicit a different response by promoting tubulin polymerization, as well as hyper-phosphorylation of a number of tubulin associated proteins that are important for cell viability, which could be attributed to their unique chemical structures. CIT-026 and CIT-223, thus, are promising therapeutic agents for, but not limited to, MPM tumors that are resistant to standard therapeutics, which warrants further *in vivo* and clinical investigation.

## 5 Conclusion

The indolyl-chalcones CIT-026 and CIT-223 significantly exert anti-tumor effects against mesothelioma cells by a dual mechanism of action: disruption of microtubule assembly and deregulation of microtubule-associated proteins STMN1 and CRMP2. The identification of tubulin as a direct molecular target of the CITs, together with the proposed binding model, will facilitate the design and characterization of next generation indolyl-chalcones aiming to increase anti-tumor activity and therapeutic index.

Both CIT compounds are highly effective, in particular against MPM cells that do not respond to current standard therapeutics, with only modest effects on normal cells, and thus represent promising therapeutic agents to impair therapy resistance and improve the treatment of MPM.

## Data availability statement

The original contributions presented in the study are included in the article/**Supplementary Material**. Further inquiries can be directed to the corresponding author.

## Author contributions

Conceptualization, GO and CK. Funding acquisition, GO. Methodology, SS, FE, CK. Investigation, SS, FE, GO, CK. Validation, SS and CK. Statistical analysis SS and CK. Molecular modeling, AFG and LS. Resources, LS, HH, JS. Data curation, SS, AH, CK. Writing - original draft preparation, SS and CK. Writing - review and editing SS, FE, LS, HH, AH, JS, GO, CK. Visualization SS and CK. Supervision AH, GO, LS, CK. Project administration CK. All author contributed to the article and approved the submitted version.
