# Supplementary material for: Indolyl-chalcone derivatives trigger apoptosis in cisplatin-resistant mesothelioma cells through aberrant tubulin polymerization and deregulation of microtubule-associated proteins
Source: Front Oncol. 2023 May 25;13:1190988. doi: 10.3389/fonc.2023.1190988 (PMC10248254; doi:10.3389/fonc.2023.1190988)
Supplement: Supplementary file 1 [file DataSheet_1.docx]

Supplementary Material

**Indolyl-chalcone derivatives trigger apoptosis in cisplatin-resistant mesothelioma cells through aberrant tubulin polymerization and deregulation of microtubule-associated proteins**

**Sophia Steinlein, Frank Essmann, Amanda F. Ghilardi, Heike Horn, Julia Schüler, Angelika Hausser, Lijun Sun, German Ott, Claudia Kalla^*^**

*** Correspondence:** Claudia Kalla: [claudia.kalla@ikp-stuttgart.de](mailto:claudia.kalla@ikp-stuttgart.de)


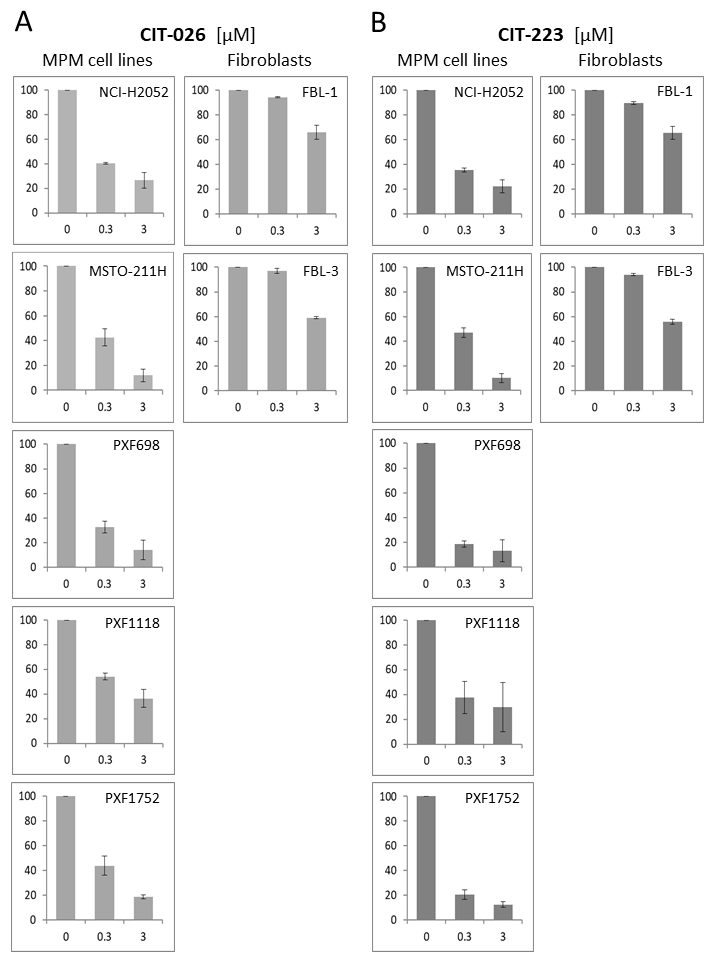


**
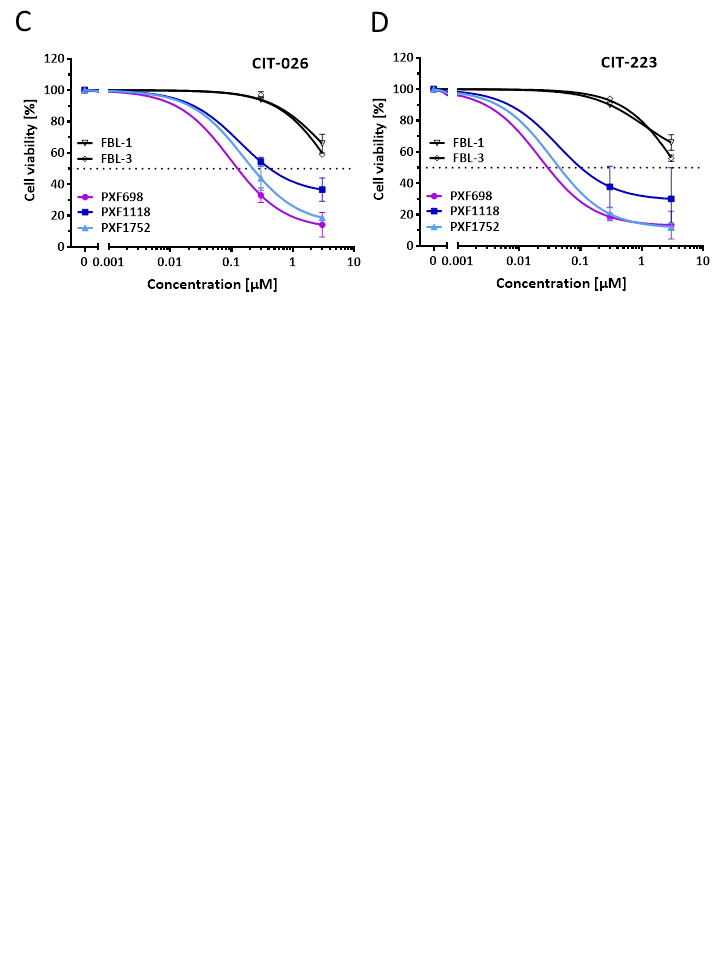
**

**Supplementary Figure 1.**

**CIT-026 and CIT-223 reduce the viability of MPM cell lines, with comparatively modest effects on non-tumor cells.** MPM cell lines and fibroblasts derived from reactive lymph nodes from two individuals (FBL-1, FBL-3) were exposed to the indicated concentrations of CIT-026 **(A, C)** or CIT-223 **(B, D)**. The viability of the cells was measured after 72h of treatment by the MTT assay. Data are shown as relative cell viability [%], normalized to cells not exposed to CITs. Mean ± SD of independent triplicate (MPM cells) or duplicate experiments (fibroblasts).

**
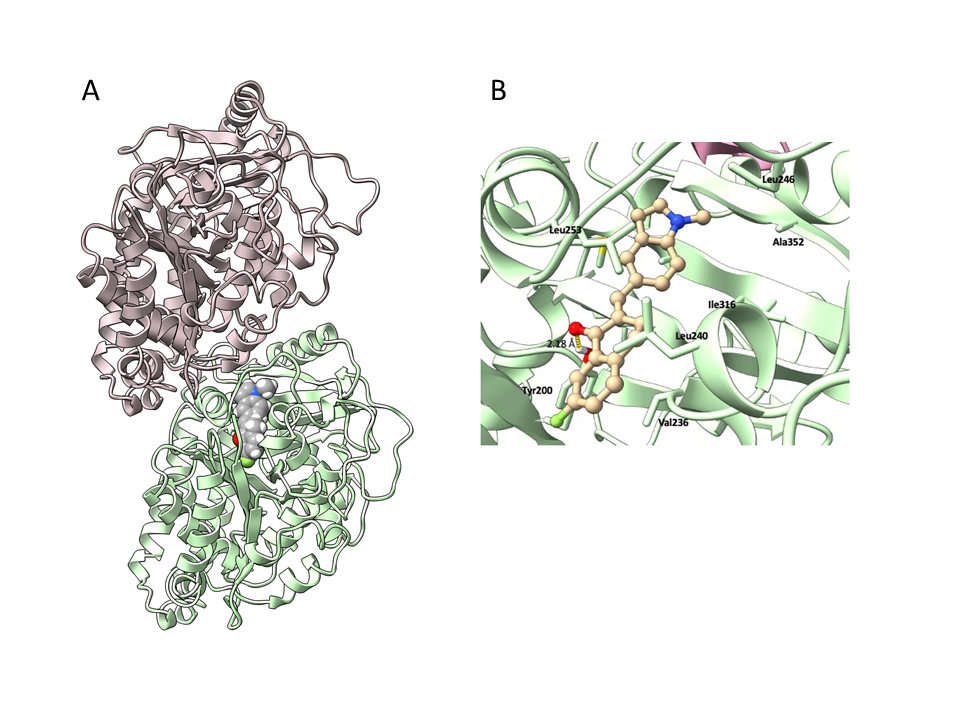
**

**Supplementary Figure 2**

**Molecular modeling of CIT-026 interaction with β-tubulin. (A)** Top docking pose (Glide Gscore: -9.09 kcal/mol) of CIT-026 in the colchicine binding site of β-tubulin. **(B)** Detailed ligand-receptor binding interactions. CIT-026 shown as spheres in grey **(A)** or as ball and stick in beige **(B)**, α-tubulin in magenta, β-tubulin in green, oxygen atom in red, nitrogen atom in blue, sulfur atom in yellow, and fluorine atom in bright green, H-bond indicated by yellow dash line.

**
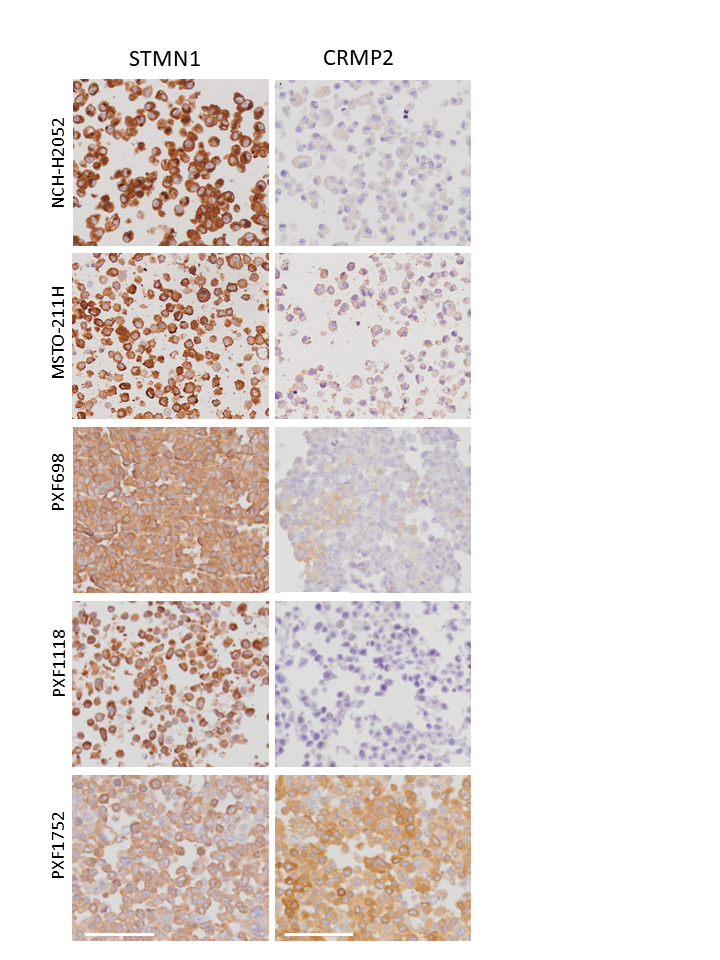
**

**Supplementary Figure 3**

**STMN1 and CRMP2 expression in MPM cells.** All five MPM cell lines expressed high levels of STMN1 whereas significant amounts of CRMP2 were only expressed in PXF1752 and MSTO-211H, determined by immunohistochemistry. Scale bar, 100 µm.

**Supplementary Table 1**

**Profile of kinase phosphorylation in mesothelioma cell line PXF698 treated with CIT-026.**

Data represent human phospho-kinase array results from PXF698 cells treated with 1 μM CIT-026 or 0.01 % DMSO for 1 h, 4 h and 24 h. Data were analyzed with the ImageJ software and are shown as the mean pixel density of duplicates within the same experiment, normalized to corresponding signals in DMSO cells.

| PXF 698 | Treatment – 1 h | | | Treatment – 4 h | | | Treatment – 24 h | | |
| --- | --- | --- | --- | --- | --- | --- | --- | --- | --- |
| **Target** | **DMSO** | **CIT-026** | **Ratio** | **DMSO** | **CIT-026** | **Ratio** | **DMSO** | **CIT-026** | **Ratio** |
| Reference | 153341 | 155133 | 1.0 | 142208 | 149526 | 1.1 | 149611 | 139424 | 0.9 |
| p38a (T180/Y182) | 4527 | 4296 | 0.9 | 2311 | 2538 | 1.1 | 4376 | 3596 | 0.8 |
| ERK1/2 (T202/Y204, T185/Y187) | 4231 | 4017 | 0.9 | 3402 | 3435 | 1.0 | 4866 | 3773 | 0.8 |
| JNK1/2/3 (T183/Y185, T221/Y223) | 7754 | 7481 | 1.0 | 7263 | 7205 | 1.0 | 7036 | 10091 | 1.4 |
| GSK-3a/b (S21/S9) | 21230 | 25111 | 1.2 | 12914 | 15838 | 1.2 | 20196 | 22575 | 1.1 |
| p53 (S392) | 17842 | 23490 | 1.3 | 15425 | 14766 | 1.0 | 30004 | 32397 | 1.1 |
| EGFR (Y1086) | 3339 | 3994 | 1.2 | 2430 | 2487 | 1.0 | 3724 | 4457 | 1.2 |
| MSK1/2 (S376/S360) | 7247 | 8675 | 1.2 | 6711 | 5734 | 0.9 | 7199 | 8494 | 1.2 |
| AMPKa1 (T172) | 8415 | 11252 | 1.3 | 10371 | 11862 | 1.1 | 10005 | 9968 | 1.0 |
| AKT 1/2/3 (S473) | 11510 | 11764 | 1.0 | 10534 | 11130 | 1.1 | 5212 | 7035 | 1.3 |
| AKT 1/2/3 (T308) | 4064 | 5627 | 1.4 | 2994 | 2915 | 1.0 | 4935 | 6710 | 1.4 |
| p53 (S46) | 6459 | 8083 | 1.3 | 4834 | 4687 | 1.0 | 10893 | 9337 | 0.9 |
| mTOR (S2448) | 3844 | 3529 | 0.9 | 3170 | 2792 | 0.9 | 3681 | 4071 | 1.1 |
| CREB (S133) | 62009 | 71341 | 1.2 | 27398 | 42677 | 1.6 | 62554 | 56910 | 0.9 |
| HSP27 (S78/S82) | 5113 | 5869 | 1.1 | 3377 | 3936 | 1.2 | 7276 | 6731 | 0.9 |
| AMPKa2 (T172) | 4781 | 4979 | 1.0 | 3242 | 3766 | 1.2 | 5667 | 5865 | 1.0 |
| b-Catenin | 5475 | 5282 | 1.0 | 5080 | 5389 | 1.1 | 4160 | 4728 | 1.1 |
| S6K (T421/S424) | 3530 | 4244 | 1.2 | 2725 | 2508 | 0.9 | 3873 | 5177 | 1.3 |
| S6K (T389) | 3899 | 4098 | 1.1 | 2639 | 2480 | 0.9 | 4484 | 4538 | 1.0 |
| p53 (S15) | 3051 | 2263 | 0.7 | 2116 | 1861 | 0.9 | 2941 | 3527 | 1.2 |
| c-Jun (S63) | 5833 | 6402 | 1.1 | 5112 | 5799 | 1.1 | 6603 | 27082 | 4.1 |
| Src (Y419) | 4933 | 4093 | 0.8 | 3310 | 3632 | 1.1 | 5721 | 5288 | 0.9 |
| Lyn (Y397) | 2897 | 2760 | 1.0 | 2464 | 2244 | 0.9 | 3568 | 3311 | 0.9 |
| Lck (Y394) | 2740 | 2678 | 1.0 | 1989 | 1785 | 0.9 | 2740 | 2765 | 1.0 |
| STAT2 (Y689) | 7144 | 7279 | 1.0 | 4021 | 4533 | 1.1 | 7983 | 8870 | 1.1 |
| STAT5a (Y699) | 2844 | 3162 | 1.1 | 2712 | 2739 | 1.0 | 3609 | 3485 | 1.0 |
| RSK1/2/3 (S380) | 3244 | 3138 | 1.0 | 2406 | 2344 | 1.0 | 3839 | 3772 | 1.0 |
| eNOS (S1177) | 2756 | 2656 | 1.0 | 1666 | 1400 | 0.8 | 2448 | 2426 | 1.0 |
| Fyn (Y420) | 3328 | 2903 | 0.9 | 2121 | 2454 | 1.2 | 3358 | 3489 | 1.0 |
| Yes (Y426) | 4267 | 4530 | 1.1 | 3139 | 3061 | 1.0 | 4295 | 5917 | 1.4 |
| Fgr (Y412) | 2636 | 2263 | 0.9 | 1749 | 1869 | 1.1 | 2526 | 2623 | 1.0 |
| STAT6 (Y641) | 6454 | 6733 | 1.0 | 5551 | 5454 | 1.0 | 5757 | 7229 | 1.3 |
| STAT5b (Y699) | 3173 | 2817 | 0.9 | 2666 | 2659 | 1.0 | 3162 | 2948 | 0.9 |
| STAT3 (Y705) | 3387 | 4134 | 1.2 | 2595 | 2620 | 1.0 | 3540 | 4854 | 1.4 |
| p27 (T198) | 3621 | 3663 | 1.0 | 2549 | 2417 | 0.9 | 3775 | 4375 | 1.2 |
| PLC-g1 (Y783) | 4015 | 3578 | 0.9 | 2297 | 2042 | 0.9 | 4266 | 4722 | 1.1 |
| Hck (Y411) | 3703 | 3575 | 1.0 | 2139 | 2880 | 1.3 | 4691 | 4163 | 0.9 |
| Chk-2 (T68) | 6382 | 8009 | 1.3 | 4553 | 4866 | 1.1 | 8116 | 22427 | 2.8 |
| FAK (Y397) | 13403 | 12277 | 0.9 | 12132 | 9120 | 0.8 | 12347 | 20268 | 1.6 |
| PDGF R beta (Y751) | 3535 | 3686 | 1.0 | 2376 | 1761 | 0.7 | 3885 | 4498 | 1.2 |
| STAT5a/b (Y699) | 5079 | 5367 | 1.1 | 3812 | 4791 | 1.3 | 4797 | 5230 | 1.1 |
| STAT3 (S727) | 3473 | 4142 | 1.2 | 2054 | 1785 | 0.9 | 4621 | 5773 | 1.2 |
| WNK1 (T60) | 19060 | 28855 | 1.5 | 12650 | 13346 | 1.1 | 20474 | 23460 | 1.1 |
| PYK2 (Y402) | 7913 | 7596 | 1.0 | 4105 | 4334 | 1.1 | 6981 | 8297 | 1.2 |
| PRAS40 (T246) | 13224 | 14326 | 1.1 | 10203 | 12069 | 1.2 | 15889 | 14827 | 0.9 |
| HSP60 | 10803 | 10683 | 1.0 | 6874 | 6550 | 1.0 | 11844 | 28472 | 2.4 |
| PBS (neg. control) | 2519 | 2460 | 1.0 | 1850 | 1488 | 0.8 | 2098 | 2214 | 1.1 |

**Supplementary Table 2**

**Profile of kinase phosphorylation in mesothelioma cell line PXF1752 treated with CIT-026.** Data represent human phospho-kinase array results from PXF1752 cells treated with 1 μM CIT-026 or 0.01 % DMSO for 1 h and 24 h. Data were analyzed with the ImageJ software and are shown as the mean pixel density of duplicates within the same experiment, normalized to corresponding signals in DMSO cells.

| PXF 1752 | Treatment – 1 h | | | Treatment – 24 h | | |
| --- | --- | --- | --- | --- | --- | --- |
| **Target** | **DMSO** | **CIT-026** | **Ratio** | **DMSO** | **CIT-026** | **Ratio** |
| Reference | 172241 | 177746 | 1.0 | 141735 | 109766 | 0.8 |
| p38a (T180/Y182) | 3688 | 4459 | 1.2 | 2710 | 2856 | 1.1 |
| ERK1/2 (T202/Y204, T185/Y187) | 5381 | 5474 | 1.0 | 6510 | 4151 | 0.6 |
| JNK1/2/3 (T183/Y185, T221/Y223) | 7286 | 8700 | 1.2 | 5699 | 6896 | 1.2 |
| GSK-3a/b (S21/S9) | 22214 | 28553 | 1.3 | 16076 | 19644 | 1.2 |
| p53 (S392) | 37361 | 40810 | 1.1 | 35954 | 37347 | 1.0 |
| EGFR (Y1086) | 4737 | 4752 | 1.0 | 4217 | 3341 | 0.8 |
| MSK1/2 (S376/S360) | 10229 | 11318 | 1.1 | 6640 | 7616 | 1.1 |
| AMPKa1 (T172) | 4398 | 5337 | 1.2 | 3514 | 4014 | 1.1 |
| AKT 1/2/3 (S473) | 5449 | 6358 | 1.2 | 4964 | 4309 | 0.9 |
| AKT 1/2/3 (T308) | 7488 | 7882 | 1.1 | 4154 | 4201 | 1.0 |
| p53 (S46) | 21428 | 25909 | 1.2 | 19435 | 20404 | 1.0 |
| mTOR (S2448) | 3577 | 4600 | 1.3 | 2974 | 3304 | 1.1 |
| CREB (S133) | 40982 | 62294 | 1.5 | 35059 | 38317 | 1.1 |
| HSP27 (S78/S82) | 4555 | 6038 | 1.3 | 3664 | 3647 | 1.0 |
| AMPKa2 (T172) | 6259 | 5032 | 0.8 | 3620 | 3974 | 1.1 |
| b-Catenin | 6422 | 7440 | 1.2 | 4039 | 3866 | 1.0 |
| S6K (T421/S424) | 4847 | 5590 | 1.2 | 2955 | 2975 | 1.0 |
| S6K (T389) | 4925 | 4863 | 1.0 | 2985 | 2762 | 0.9 |
| p53 (S15) | 4796 | 6421 | 1.3 | 4283 | 3967 | 0.9 |
| c-Jun (S63) | 7451 | 9153 | 1.2 | 6024 | 16790 | 2.8 |
| Src (Y419) | 5029 | 5183 | 1.0 | 4138 | 3919 | 0.9 |
| Lyn (Y397) | 3608 | 3650 | 1.0 | 2091 | 2206 | 1.1 |
| Lck (Y394) | 3646 | 3555 | 1.0 | 2101 | 1963 | 0.9 |
| STAT2 (Y689) | 7780 | 8635 | 1.1 | 5614 | 6253 | 1.1 |
| STAT5a (Y699) | 4348 | 4177 | 1.0 | 2539 | 2178 | 0.9 |
| RSK1/2/3 (S380) | 3941 | 4226 | 1.1 | 2598 | 2859 | 1.1 |
| eNOS (S1177) | 2782 | 3125 | 1.1 | 2047 | 1701 | 0.8 |
| Fyn (Y420) | 3759 | 4168 | 1.1 | 2513 | 2400 | 1.0 |
| Yes (Y426) | 5338 | 5211 | 1.0 | 3603 | 3198 | 0.9 |
| Fgr (Y412) | 3368 | 3726 | 1.1 | 2617 | 1933 | 0.7 |
| STAT6 (Y641) | 7694 | 8734 | 1.1 | 5328 | 4947 | 0.9 |
| STAT5b (Y699) | 3846 | 4181 | 1.1 | 3122 | 2315 | 0.7 |
| STAT3 (Y705) | 5454 | 5835 | 1.1 | 3214 | 4329 | 1.3 |
| p27 (T198) | 4977 | 5302 | 1.1 | 3700 | 3108 | 0.8 |
| PLC-g1 (Y783) | 4385 | 4449 | 1.0 | 3358 | 2727 | 0.8 |
| Hck (Y411) | 4630 | 6215 | 1.3 | 3368 | 4141 | 1.2 |
| Chk-2 (T68) | 6636 | 7293 | 1.1 | 5022 | 5642 | 1.1 |
| FAK (Y397) | 15207 | 18574 | 1.2 | 11639 | 13844 | 1.2 |
| PDGF R beta (Y751) | 4501 | 5209 | 1.2 | 3223 | 2699 | 0.8 |
| STAT5a/b (Y699) | 5221 | 6861 | 1.3 | 3557 | 3585 | 1.0 |
| STAT3 (S727) | 19095 | 28889 | 1.5 | 19800 | 27090 | 1.4 |
| WNK1 (T60) | 12008 | 15708 | 1.3 | 8865 | 9493 | 1.1 |
| PYK2 (Y402) | 9327 | 9056 | 1.0 | 6009 | 5386 | 0.9 |
| PRAS40 (T246) | 9268 | 10128 | 1.1 | 7732 | 7642 | 1.0 |
| HSP60 | 10216 | 18258 | 1.8 | 10500 | 14476 | 1.4 |
| PBS (neg. control) | 2846 | 3208 | 1.1 | 1945 | 1887 | 1.0 |
